# Chicago Medical Society

**Published:** 1888-03

**Authors:** 


### Society Reports.

### CHICAGO MEDICAL SOCIETY.

STATED MEETING, November 21, 1887.

The President, W. T. Belfield, M. D., in the chair.

Dr. L. L. McArthur read a paper on

### THE PRESENT STATE OF TETANUS.

Tetanus is divided into acute, subacute and chronic. Every case of tetanus has, as a primary factor, a traumatism which may be no more than the prick of a hypodermic needle or may be the crushing of a limb. Blacks are said to be more liable to tetanus than whites. The occupation must also be taken into consideration, hostlers, gardeners, etc., being especially susceptible to the disease. It is claimed that tetanus is always infectious and never spontaneous. The case was mentioned of a New York surgeon, who, when making a post-mortem examination of a horse that had died of tetanus, accidentally scratched his finger and shortly after died of the same disease. Experiments have been made by which a microörganism, cultivated from garden soil and introduced under the skin of animals, produced tetanus, and muscular tissue obtained from a case of tetanus introduced under the skin of lower animals, reproduced the disease. Out of 16,000 cases of foot injuries during the war of the rebellion, fifty-seven resulted in tetanus. In 12,000 hand injuries not a single case of tetanus was reported. In the acute stage the termination is always fatal by the end of the fourth day from spasm of the larynx or heart failure. In the subacute there are 50 per cent. of recoveries. The chronic form terminates favorably in from thirty to sixty days. In the treatment nervous sedatives, combined with absolute quiet and rest, yield the best results. The bromides and opium seem to have the most beneficial effect.

Dr. A. E. Hoadley: I have been very much interested in this paper, and while I am not prepared to contribute anything in the way of theory as to the pathology or etiology of this disease, I am surprised to see how few cases have been cited, but seventy-seven having been collected; then again I am surprised to learn that fifty-four out of the seventy-seven recovered. It is probable that this is a very small percentage of the whole number; it seems to me that it is a common disease. I have had ten cases of traumatic and four cases of infantile tetanus in my practice.

Dr. N. P. Pearson: Yesterday I received a medical paper from Copenhagen, in which I saw a report of a case of infantile trismus. They have found bacteria there. They took a part of the navel and produced infection from it; they infected mice and guinea pigs, and they died immediately of tetanus.

Dr. Frank Billings: It may be interesting to cite two or three cases that have come under my observation, especially as to the treatment. While in Cook County Hospital, there were, I remember, two cases of especial interest to me: One was a man about 30 years of age, who had received an inoculating wound from a pitch-fork through the foot, passing downward from the dorsum. He did not come to the hospital until trismus had commenced. The wound in the foot was suppurating then, and Dr. Meacher, of Portage, Wis., who was house surgeon, opened up the local wound and dressed it antiseptically. Tetanus developed, and on the third day after he entered the hospital the sciatic nerve of that side was stretched. It did not have a good influence, and three days afterwards the crural nerves were stretched, and after that the sciatic nerves were again stretched without marked effect, good or bad. At the same time he was put upon morphine and chloral; afterwards he was given the treatment recommended by Dr. Gunn—hypodermic injections of physostigma. We began with $\frac{1}{8}$ of a grain, repeated every three hours, and gradually increased the amount until the man took 3 grains every dose, of the stock which was in the hospital, without a perceptible effect. The stock giving out, a new supply was obtained, and the nurse went on giving 3 grains of the new supply. I was called suddenly to see the man and found him with all the characteristic symptoms of physostigma poisoning—pin-point pupils and rapid, weak pulse, œdema of the lungs with rapid superficial breathing. I gave the usual antidotes, atropine and digitalis, and he soon recovered; the first thing he did was to assume the tetanus position—ophisthotonos. He eventually recovered, but he never regained his strength of mind—he was simple, had a peculiar laugh and talked a great deal; before he had been a silent man, but he became very garrulous and was the clown of the ward. He disappeared from the hospital and I lost sight of him. Another case of interest occurred while I was house surgeon. A man was brought to the hospital with a simple fracture of one tibia; in two weeks trismus suddenly developed after a chill. On examining the man, I detected fluctuation over the seat of fracture and the bones were freely movable. Dr. Isham was the attending surgeon. We made a compound fracture of it and drained the wound thoroughly, and although the trismus remained for several days, the man ultimately recovered with perfect union of the bones. Another case—which is not of special medical interest—was a boy who received a crushing injury of one leg. I amputated the leg in its upper third; the flaps united very well, but about the seventh day he was taken with a chill, and trismus commenced, and then tetanus developed. The friends of the boy consented to have the sciatic nerve stretched, but when we attempted to have it done—had the boy carried into the operating room—the warden and chairman of the hospital committee interfered and commanded us to take the boy back; as a result, the old medical board stepped out of the County Hospital. We afterwards stretched the nerve and the boy recovered—making three recoveries from tetanus during my service in the hospital. Several other cases of tetanus occurred from 4th of July pistol wounds and death followed in every one. The temperature just before and after the death was the same as in all hydrophobic patients—it rose toward death, and my thermometer, which registers 110° F., would not register it.

Dr. J. A. Robison: Last summer, during my service in the County Hospital, there was a case of tetanus developed in my ward. The man was brought in May with what was supposed to be cerebral meningitis; he had a high temperature, face flushed and of a purple hue. He was easily excited, and in a day or two we noticed symptoms of tetanus, trismus and ophisthotonos occurring during the progress of the case. During the January previous, he had received a wound from a buzz-saw in the second and third fingers of the right hand. He was sent to the surgical side and these fingers were amputated. For several days it was a question whether or not he would recover, but he finally did recover, and the last I saw of him he was walking about the ward, although he was insane. I think the amputation probably saved his life, because these fingers were undoubtedly a source of irritation, as he would have tetanic spasms only when these fingers were touched. This would seem to indicate that it is possible that old wounds act as an exciting cause of tetanus.

Dr. L. L. McArthur: In regard to the frequency of recoveries in the cases reported, of fifty-four out of seventy-seven, I would say that it is probable that the cases which terminate successfully find their way into the medical journals, while those that terminate fatally are not so frequently published, and this will explain the apparent excess of cures over deaths. To say that the disease is a frequent one I think is a mistake—it is one of the rare diseases, although one sees it occasionally in practice. During the entire war there were reported 28,000 wounds of the hands and feet, and of these only fifty-seven resulted in tetanus (*i. e.*, 1.2 of 1 per cent.), thus showing that the disease is not very frequent. I am glad to learn that tetanus has been proven to be of an infective nature, because this offers the best explanation of the causation of this disease that has yet been given. Several eminent authorities have defined tetanus as a disease of uncleanliness, which would rather point toward its infectious character. In the case reported by Dr. Robison, he admits that there was a meningeal doubt in the case, and I think in many cases of so-called tetanus, investigation would prove them to belong to a different class of nervous diseases. One point I think well worthy of emphasis, that is the continuance of the treatment long after all the symptoms of tetanus have disappeared, and I very much regret, in the last case which I had, that I diminished the amount of medicine that the man was taking during an apparent improvement in the course of the disease. Several French authorities also urge this point.

Dr. W. T. Belfield reported a case of

NEPHRO-LITHOTOMY.

James F., 37 years old, gives the following history: Some fifteen years ago he suffered for several months from great irritability of the bladder, urinating very frequently and with considerable pain. On several occasions he passed blood. Was treated at that time for "cystitis." These symptoms gradually disappeared; at intervals thereafter he suffered from dull pain in the loins, often extending upward to the chest and sometimes incapacitating him for work. For the past two years pus had been seen in the urine constantly in varying quantity. At times the amount would be small for a few days, during which time the pain in the side became severe, then a large quantity of pus would be discharged while the pain diminished. After treatment for various complaints he came, about a year ago, under the care of Dr. Frank Billings, who discovered an increased area of dullness over the left kidney, and at times a distinct, though slight, swelling in the loin. Recognizing that the left kidney was the seat of suppuration, Dr. Billings endeavored to ascertain the cause therefor. Tuberculosis was almost certainly excluded by repeated examinations of the pus for the characteristic bacilli with negative result. As renal calculus seemed the most probable diagnosis, Dr. Billings referred the patient to me for operation. After thorough examination and observation, this seemed to me also the most plausible diagnosis, though many of the acute symptoms of calculus did not then exist. The right kidney could be felt through the abdominal walls and seemed normal, but previous to the operation, I endeavored to demonstrate its integrity by closing the ureter of the one under suspicion by means of Silbermann's catheter. The instrument caused so much pain, however, that the result was unsatisfactory. June 26 last, in the presence of Drs. Billings, Hosmer and Tilley, I explored the left kidney through an oblique lumbar incision. The organ was found to be enlarged and flabby. Upon incising the cortex an explanation of this condition was found in hydro-nephrosis, only a shell of the renal tissue half an inch thick remaining. The cause of hydronephrosis was found in a calculus of conical shape some two inches in length, its smaller end impacted in the ureter so tightly as to require some force for its extraction. A few loose flakes were afterwards removed from the pelvis and ureter. Recovery ensued without notable event. On the fourteenth day the patient visited his place of business. In two months his weight increased from 105 to 123 pounds, and all morbid symptoms had disappeared, except that some pus was still present in the urine. Latterly this has become so slight in amount as to be discoverable only with a microscope. Presumably the shriveling of the distended kidney is about accomplished. The stone is an oxalate or mulberry calculus and weighed 224 grains.

Dr. Belfield also reported a case of

CANCER OF THE PROSTATE.

Hans J., aged 48 years, was admitted to the County Hospital, suffering from unduly frequent and somewhat painful urination, symptoms which had existed for some three months. On two occasions blood had been observed in the urine. Examination *per rectum* showed a slightly enlarged, though symmetrical, prostate, along the centre of which, instead of the normal depression, was a slight protuberance. By the microscope we found in the urine a few blood corpuscles. A tumor of the prostate, either epitheliomatous or malignant, was diagnosed. The latter seemed more probable because the quantity of blood in the urine was altogether too slight for the benign variety. Efforts to entangle and bring away shreds of the supposed growth in the eye of a metallic catheter failed. May 7 the bladder was explored by supra-pubic incision, and malignant, villous growth was found growing over the left side of the prostate, the villi projecting an inch or more into the bladder. These were removed with forceps and spoon and the base cauterized. Recovery ensued without notable event, and for eight weeks the renal function seemed almost normal; then the former symptoms began again and steadily increased in severity. The growth finally entirely filled the bladder and involved the abdominal wall, producing a small fistulous opening, and constituting a tumor which could be plainly outlined above the symphisis and through the rectum, apparently filling the pelvis. Death occurred October 8. Only the glands in the immediate vicinity of the bladder were found to be carcinomatous and no secondary growths in other organs were discovered.

Dr. Elbert Wing: There is a point in regard to the exclusion of tuberculosis by the examination mentioned that is perhaps of interest in this connection. A gentleman, who served for two years as interne at Tübingen, told me that they had a case there which they thought was tuberculosis of the renal tract, and that they made between sixty and seventy very careful examinations of the sediment of the urine for the bacilli of tuberculosis, but found none. At the autopsy they found tubercular ulcers in the kidneys, both ureters, bladder and urethra.

Dr. Elbert Wing exhibited a specimen from a case of

TYPHOID FEVER.

I have here some fresh pathological specimens obtained at the County Hospital to-day. The first is a portion of the intestine of a man that died of typhoid fever. It shows the swelling and tumefaction of the glands very nicely. There are also a few ulcers. There is a point of interest about the case, aside from the typhoid fever, which was the involvement of the respirations, averaging between 40 and 60, pulse 120, temperature 101°. When the case came to the post-mortem table, the lungs were found to be quite extensively œdematous.

Dr. Wing also exhibited specimens of a case of

CIRRHOSIS OF THE LIVER WITH MARKED ASCITES.

The man had been tapped a number of times and finally died in the hospital from œdema of the lungs. He also had a scrotal hernia, and in addition to that there was an hydrocele of the same side. The omentum was attached to the anterior surface of the sac perhaps two inches from the external ring, and throughout the omentum there are wide bands of connective tissue which have formed through chronic passive hyperæmia. All of the connective tissue about the liver was in a similar condition. The hydrocele was the size of a goose egg and has been incised. The testicle is perhaps one-third smaller than one would expect to find it. The organ of principal interest, however, is the liver. All that need be said about the history of the case is that the man enjoyed the *sobriquet* of "Champagne Charlie." It is as handsome a specimen of gin-drinker's liver as one ever sees. One can see the little projecting islands of tissue, which are almost small enough to give the surface of the liver the appearance of granulation tissue. By way of emphasis of the difference between this kind of cirrhosis and the so-called biliary cirrhosis, of which there is not as yet much definitely known by the profession at large, I have taken the trouble to bring some slides of this and of biliary cirrhosis. They demonstrate very beautifully the difference in the way in which the connective tissue is distributed throughout the organs. In diffuse cirrhosis it is peripheral to the lobule, and surrounds nearly all of the lobules in any given field, but the connective tissue in biliary cirrhosis shows itself first around the gall-ducts as little circumscribed islands clearly defined. There seem to be two forms of biliary cirrhosis, one with and one without hypertrophy of the liver, the former usually occurring as an acute disease in cases of hard drinkers.

